# Antecedents for crafting a sense of coherence among healthcare employees

**DOI:** 10.3389/fpsyg.2025.1376472

**Published:** 2025-11-12

**Authors:** Ellen Jaldestad, Lotta Dellve

**Affiliations:** 1 Division of Ergonomics, Department of Biomedical Engineering and Health Systems, KTH Royal Institute of Technology, Stockholm, Sweden; 2 Department of Sociology and Work Science, University of Gothenburg, Gothenburg, Sweden

**Keywords:** health promotion, job crafting antecedents, sense of coherence, sustainable work, system perspective, healthcare employees

## Abstract

Job crafting–an active form of job redesign–has been widely studied within healthcare in recent years, possibly because of its many positive outcomes in this context. Job crafting can be described as actions to redesign a current work situation to increase the fit of a person’s abilities, resources, and desires. Among the positive consequences of job crafting are employee well-being, work engagement, and a productive workforce. The Swedish healthcare sector struggles with challenging work environments and staff shortages, and health-promoting activities can thus be particularly important for employees’ wellbeing. To identify antecedents of job crafting that can promote well-being within public healthcare, semi-structured interviews were conducted with employees and managers in five different healthcare departments. Departments with daytime activities only and 24/7 operations were represented in the study. On an individual level, being a driven person as well as focusing on the patient were found to precede health-promoting job crafting. Being a driven person indicates an ability to deal with restrictive work conditions, such as a lack of support among colleagues and managers, for those who choose to craft their jobs. Working autonomously or in cross-professional teams were contextual antecedents that led to a more comprehensible work situation and different degrees of perceived freedom in work. Utilizing these degrees of freedom through job crafting strategies with the patient’s best interests in mind led to a work situation that was considered more manageable and meaningful. When planning how to facilitate health-promoting job crafting among healthcare employees, it is recommended to keep in mind that job crafting antecedents seem to interplay on different organizational and individual levels.

## 1 Introduction

Sweden has a long tradition of focusing work environment and work-related well-being through extensive regulations and cooperation with trade unions (Dellve et al., 2024). Working conditions have in general been improved during the last decades, however, the female-dominated healthcare sector has fallen behind (Dellve et al., 2024). The healthcare sector in Sweden and in the north European countries, struggle with challenges such as stress and physical workload, high percentages of sick leave, staff shortages, and retention (Dellve et al., 2024; Felder et al., 2024; Fogelberg Eriksson and Halvarsson Lundqvist, 2024). Current statistics show that psychosocial diagnoses count for 50%, and musculoskeletal diagnoses for 15% of long-term sick leave among nurses in Swedish public healthcare, with a total risk of long-term sick leave around 10 out of 1,000 people. Among assistant and dental nurses, the distribution of diagnoses is 37% psychosocial diagnoses and 29% musculoskeletal diagnoses, and the total risk of long-term sick leave is around 11 out of 1,000 people. The risk of long-term sick leave is higher among women in all groups (AFA Insurance, n.d.). To respond to these work-related challenges, as well as the complexity of work, there is a general need for a holistic and health-promoting focus in work (Dellve and Eriksson, 2017). This paper contributes to required knowledge by identifying antecedents for health-promoting job crafting at workplaces and among individuals, who are recognized for applying such strategies despite their challenging work organization context.

Health-promoting work can be framed within the salutogenic theories on sense of coherence (SOC), developed by Antonovsky (1987). With a strong SOC, people perceive their situation as comprehensible, manageable, and meaningful (Antonovsky, 1987, 1996). A work situation that is perceived comprehensible is structured, predictable, and explicable, and employees understand structures and processes within the organization. A manageable work situation provides enough social and structural resources to handle challenges and demands. Meaningfulness in work is connected to employees’ motivation through, for example, colleagues, professional pride, and personal development (Antonovsky, 1987, 1996; Michele Masanotti et al., 2020; Vaandrager and Koelen, 2013). Job crafting was originally described as employees’ actions to redesign their current work situation, by changing the physical or cognitive boundaries of the job (Wrzesniewski and Dutton, 2001). A person who conducts job crafting, a job crafter, can, for example, add new and challenging tasks, cultivate supporting relations with colleagues, or change their view of certain tasks (Wrzesniewski and Dutton, 2001). The purpose of job crafting is to optimize the fit between job demands and resources on the one hand, and personal goals on the other (Tims and Bakker, 2010; Wrzesniewski and Dutton, 2001). Job crafting has in general been connected to positive work-related and health-promoting outcomes for both job crafters and their employers. Among these are work engagement and motivation, job satisfaction, job performance, and resilience (e.g., Bakker and Demerouti, 2017; Hakanen et al., 2017; Jaldestad et al., 2025; Tims and Bakker, 2010).

## 2 Background

### 2.1 Job crafting antecedents

Previous research has identified several conditions that seem to facilitate job crafting in general. First of all, employees must be motivated to craft (Wrzesniewski and Dutton, 2001), and since job crafting is a bottom-up driven process, individual traits such as a proactive personality can enhance such activities (Bakker et al., 2012; Ghazzawi et al., 2021; Rudolph et al., 2017). Among healthcare employees, energy and work engagement, work experience and level of skills have also been found to facilitate job crafting (Chang et al., 2020; Ghazzawi et al., 2021; Hakanen et al., 2017; Harbridge et al., 2022; Mayson and Bardoel, 2021; Roczniewska and Bakker, 2021; Sahay and Dwyer, 2021). Apart from these positive characteristics, Hakanen et al. (2017) found that workaholism and burnout were positively related to crafting among dentists, more specifically to increasing structural resources and challenging job demands and decreasing hindering job demands in work.

Since individuals act within a given work context, there is also an organizational perspective on job crafting antecedents; organizational structures and workplace characteristics can facilitate or hinder job crafting. Autonomy and task independence have, for example, been found to promote job crafting in general (Felder et al., 2024; Lazazzara et al., 2020; Tims and Bakker, 2010). A supportive work context (e.g., support from colleagues and managers, social capital, people-oriented leadership approaches, empowerment, and supportive job design) can promote approach-oriented job crafting, such as adding extra tasks and positively reframing roles, with the positive outcomes mentioned above (Audenaert et al., 2020; Esteves and Pereira Lopes, 2017; Harbridge et al., 2022; Jutengren et al., 2020; Lazazzara et al., 2020; Pan et al., 2021). Social support among colleagues has also been found to moderate the interaction between empowering leadership and job crafting among healthcare employees (Audenaert et al., 2020). Hierarchical structures have been connected to job crafting; the higher the rank position, with high levels of formal autonomy and power, the more space for prioritizing tasks (Palm and Eriksson, 2018). Higher-rank employees perceive job crafting challenges as being within their expectations of how work should be conducted, whereas lower-rank employees tend to put greater emphasis on their job description, and what others expect from them (Berg, Wrzesniewski, et al., 2010). A lower degree of engagement–for instance, because of fewer perceived opportunities, and working only for the money–was early found to reduce job crafting activities (Wrzesniewski and Dutton, 2001). Other identified job crafting barriers include lack of support, heavy workload, and staff shortage (Harbridge et al., 2022). Constraining work contexts, with little support and resources, have been associated with crafting strategies such as reducing the number of tasks and relations, with negative outcomes for the job crafter (e.g., stress and regrets) (Lazazzara et al., 2020). From an employer’s point of view, job crafters may jeopardize company performance if, for example, they neglect troublesome tasks or clients (Berg, Wrzesniewski, et al., 2010). Job crafting does not appear in a vacuum, or by itself. Instead, individual conditions interact with conditions of professional roles, and organizational structures (Felder et al., 2024; Lazazzara et al., 2020).

### 2.2 Job crafting and work-related sense of coherence

Palm and Eriksson (2018) interviewed managers and employees in different sectors about their strategies to master intense work, and found both active and cognitive salutogenic crafting strategies, including prioritizing work and asking colleagues for help, as well as redifining their thoughts about work for increased manageability in work. There were examples of individual strategies as well as strategies that involved others (i.e., managers and/or colleagues). In relation to SOC, Lichtenthaler and Fischbach (2016) found that promotion-focused job crafting (i.e., increasing social and structural job resources, and increasing challenging job demands) was positively related to work-related SOC, whereas prevention-focused job crafting (i.e., decreasing hindering job demands and reducing tasks) was negatively related to work-related SOC among older police employees (aged 54–62 years). Previous research among healthcare employees indicates that higher SOC follow more understanding of the overall work context. So too do working regularly compared to irregularly (Michele Masanotti et al., 2020). A strong SOC can contribute to maintaining health and well-being at work and to choosing proactive crafting strategies to manage stress in demanding work environments in general (Betke et al., 2021; Palm and Eriksson, 2018). Among healthcare employees, meaningfulness and comprehensibility in work have been connected to positive mental health (Mantas-Jiménez et al., 2024). A recent study including Swedish healthcare employees indicates the importance of salutogenic resources in supporting newly graduated healthcare professionals. In the study, a strong SOC and higher score on meaningfulness were found among employees who also indicated that they were sure to stay in their new profession. SOC and its’ dimensions were all connected to the respondents’ general health (Larsson et al., 2025).

Although it has not been specifically expressed, some studies have found associations between job crafting and the three components of SOC within the healthcare sector. For instance, a recent study conducted during the COVID-19 pandemic found that job crafting strategies among general practioners increased meaning in work, when being able to manage work under this unfamiliar and challenging period (Månsson Sandberg et al., 2024). In addition, Felder et al. (2024) found that job crafting among nurses led to a more comprehensible and manageable work situation when autonomy in work was provided–nurses were encouraged to develop their own roles and were in this able to take a helicopter view of their department and how to conduct their tasks.

### 2.3 Focus and aim of this study

The previous findings presented above suggest that the work context and what is going on during the workday contribute to individuals’ job crafting strategies and their consequences, and there is not one condition that automatically leads to job crafting for all individuals. They also indicate that conditions that facilitate job crafting cannot be explained in one simple way; instead, job crafting antecedents seem to interplay on different levels (Lazazzara et al., 2020; Park and Park, 2023; Zhang and Parker, 2019). Thus, leadership approaches and daily work management interact with workgroup characteristics (e.g., social support), as well as job characteristics (e.g., job autonomy and task independence), and individual preconditions (e.g., being proactive and having experience in work) (Lazazzara et al., 2020; Park and Park, 2023). A system perspective on job crafting antecedents can broaden the understanding of conditions that either facilitate or hinder job crafting among employees (Dellve and Eriksson, 2017). In this study, different work-related levels were therefore included in the interviews and analyses. Individual factors (e.g., personal conditions) were combined with characteristics on the workgroup level (e.g., group climate and social capital) and workplace level (e.g., perceived leadership approach and job characteristics). In this study, the focus was on job crafting strategies that contributed to work engagement, motivation, and job satisfaction, and on developing strategies to manage challenges and demands. This is considered to relate to individuals’ work-related well-being and is further referred to as *health-promoting job crafting* in this article. There was a general focus on identifying such strategies in this study, and a particular focus on identifying their precursors. Against this background, this study aimed to identify antecedents of health-promoting job crafting among healthcare employees. A further theoretically driven aim was to interpret the inductively analyzed antecedents and relate them to work-related sense of coherence.

## 3 Method

This study was conducted as part of a larger research project within Swedish public healthcare. Data was collected with a broad aim of well-being at work. We used a qualitative study design to start answer the aim with inductive approach and thereafter relate the findings to a, for the concept and findings, relevant earlier theory (in discussion section) (Karlsen et al., 2021). To achieve experiences and observe related handling, interviews were conducted with different healthcare professionals, as well as observations at respondents’ workplaces. We also analyzed internal material on work environment development within the participating departments. The interviews with the healthcare employees have been used in another study aimed at exploring different job crafting strategies that healthcare employees engage in to promote well-being in work. The different aims of the studies have focused on different parts of the collected data.

### 3.1 Sampling and respondents

The study was conducted in five departments within Swedish public healthcare, located in three different regions. Professional networks of the research team were used to recruit participating departments. In line with the overall aim of the research project, departments that were known to actively work with health-promoting processes where employees were involved were selected. Departments with daytime activities only (dental clinic, surgery department, occupational therapy), and 24/7 operations (surgery care department, radiology department) were represented in the study. However, due to availability, there were no respondents working only, or mostly, night shifts among the informants.

Clinic managers and ward managers were initially contacted with an invitation to participate in the research project. To provide empirical data from a range of professions and work experience as broad as possible, respondents from different professional groups, and with varying lengths of work experience, were thereafter requested to participate by those managers who accepted the invitation. Employees were contacted by their managers with an invitation to participate in the project sent either by e-mail or through personal contact. Participation was voluntary, albeit known to their closest manager. To broaden the sample and data material, managers were in some cases contacted with specific requests for employees from specific professional groups or lengths of work experience. This resulted in 21 informants, of which 16 were employees and five were managers: one dental nurse, four assistant nurses, five registered nurses, six occupational therapists, one section leader, one clinic manager, and three ward managers. Seventeen respondents were women and four were men, and the work experience in the current profession ranged from 6 months to 43 years. Among the managers, experience in managing positions ranged from 4 years to about 10 years (see Table 1).

**Table 1:** Sample characteristics.

Professional role	Years in current professional role	Gender	Professional role	Years in current professional role	Gender
Dental nurse	43	Female	Occupational therapist	20	Female
Assistant nurse	40	Female	Occupational therapist	8	Female
Assistant nurse	14	Female	Occupational therapist	7	Female
Assistant nurse	8	Female	Occupational therapist	2,5	Male
Assistant nurse	4	Male	Occupational therapist	2,5	Female
Registered nurse	18	Female	Occupational therapist	0,5	Male
Registered nurse	8	Female	Section leader	10	Female
Registered nurse	4	Male	Clinic manager	5	Female
Registered nurse	2,5	Female	Ward manager	5	Female
Registered nurse	1	Female	Ward manager	4	Female
			Ward manager	4	Female

Both managers and employees were included in the data collection to provide data from their different perspectives. When interviewing the employees, it was of interest to explore the occurrence and nature of job crafting strategies, as well as the perceived conditions that facilitated such strategies. The managers were included in the data collection to provide their view of the working contexts, as well as their intentions to promote job crafting strategies among their employees (e.g., intended leadership approach).

### 3.2 Data collection

Individual semi-structured interviews were conducted until there was a perceived breadth of informants and depth in the material (Low, 2019; Scanlan, 2020). The interviews were conducted at the respondents’ workplaces during working hours, in closed and separate rooms, and the interviewees were allowed to take the time for their interviews without being disturbed. All interviews were recorded and lasted about 1 h. Two of the informants (two registered nurses) were interviewed twice due to an organizational change, 1 year apart, while the other informants were interviewed on one occasion only. The first author of this article (a female doctoral student) conducted all interviews except those with the occupational therapists, which were conducted by a female master’s student. Data was collected from 2017 to 2019.

The questions in the interview guides were developed in line with the overall aim of the current research project, and from the job crafting literature presented above. Due to the overall purpose of the research project, the interviews were quite extensive in scope, and included questions to capture both job crafting strategies and their antecedents, as well as questions about the workgroup and perceived leadership. For this study, questions that concerned job crafting and health promotion in work were the main focus, which covered the following areas: *background information* (e.g., professional role, work experience); *preconditions and strategies for job crafting* (e.g., perceived opportunity to change and develop within current work settings); *leadership approach* (e.g., perceived, and intended leadership styles); *workgroup context* (e.g., trust, roles, maturity); *learning from challenges and hindrance*s (e.g., strategies to handle obstacles, outcomes); *perceived and desired resources at work* (e.g., relational and structural resources). These focus areas were the same when interviewing employees and managers, while the questions asked within the areas differed slightly between these two groups of respondents, as well as between the two interviewers.

When visiting the various departments, pictures were taken to exemplify practical tools used to develop the work environment (e.g., visualizing boards, mood gauge). In addition, observations were conducted in two departments–the surgery care department and the dental clinic–to provide information about the respondents’ work settings. During these observations, notes were taken and daily work was followed during clinical work, common breaks, and one staff meeting in the dental clinic. In the surgery care department, work was followed from the corridors and common spaces and small talk with the employees. Pictures and notes from the visits and observations were included in the analyses in terms of broadening the understanding of the working contexts.

### 3.3 Ethical consideration

The Ethical Review Board in Stockholm approved the study (Dnr. 2014/1883–31/5), and informed consent was applied. Before agreeing to participate, all respondents were informed both verbally and in a written text about the study, as well as about their right to stop the interview at any time, without any data being saved. Then each signed a consent form. Since managers were involved in recruiting the informants, it was important to ensure that the latter participated voluntarily, and without the risk of being negatively affected by their participation (Resnik, 2016), as well as to ensure their confidentiality when managing the collected data (Vetenskapsrådet, 2017).

### 3.4 Data analysis

The recorded interviews were transcribed verbatim and thereafter imported into NVivo–in which the interview transcripts and pictures from the observations were stored, organized, and analyzed. As mentioned above, the material from the visits and observations was used to broaden the understanding of the working context and was not analyzed further (i.e., not coded in the further analyses of the interviews). To identify and explore the antecedents of health-promoting job crafting, a framework of reflexive thematic analysis was chosen (Braun and Clarke, 2006, 2021). The interview material was analyzed abductively: the interview transcripts were initially coded inductively by the first author of this article, with an open approach toward the data. When moving forward, codes and initial themes of health-promoting job crafting antecedents were interpreted from our pre-understanding of the theories of job crafting and health promotion presented above (Braun and Clarke, 2006, 2021; Graneheim et al., 2017; Karlsen et al., 2021). The analyzing process was iterative and reflective, as suggested by Braun and Clarke (2006, 2021), and included recurrent discussions between the authors. Initial themes were revised with joint rounds of interpretations, regularly tested on the empirical data, and adjusted before agreeing on their final form and naming them. A summary of the analysis process is presented in Table 2.

**Table 2:** Flowchart of the analytical process.

Examples from the transcripts	Examples of initial coding	Themes: job crafting antecedent
*I saw the need, both in the department and in the entire clinic, and then I try to keep myself updated through various conferences and training courses. And there you hear that there are similar services. … I designed it, came to an agreement with the clinic manager, went to my boss and said that I wanted to work this way. I showed that it was needed. And then I got it.* *I think it depends on the person. Because I am like that. I look for a challenge. I want to do things, I see, I want to make and implement improvements, but if I do not get a response, if I do not get permission, then I’m not interested. Then I’ll move on, of course. It’s both parts.*	Identifying and showing a need Keeping themself updated Designing their own work Looking for a challenge in work – wants to develop regardless of managerial support	Being a driven person
*We are not all the same here, but we complement each other. Many of those who still work here are completely different, but we are very different in our work. However, we share the same basic idea: we are here for the patient. We are working toward the same goal. …* *You have to think, or at least I always think about whether I want my family here. I take care of them as if it’s someone’s mother that I am taking care of, or someone’s daughter, or someone’s. I think it is about respect and that you want the same thing.*	Being different but working toward the same goal Being there for the patient Caring for patients like their own family Being respectful to patients and their families	Focusing on the patients’ wellbeing
*It does not fill a whole day, in my opinion. I have tried to make two home visits a day but it does not really work, my head gets chaotic. If one is tough and demanding, you do not have the energy for another one in the afternoon and you never know what to expect, so it is not possible. But in terms of time, it does not fill a day. Then there’s a bit of R&D work in the afternoon. Then I can have meetings like this one, in the late afternoon, when I have plenty of time for meetings, student supervision, or things like that. That’s how it can be, that’s my normal daily rhythm.* *… you have to think about how they cope with everyday life and so on, but it’s not up to me to solve that. … I have close contact with the physicians here, they sit in the corridor next door. They help me a lot and I help them and so on. So that exchange is great fun. And … you always get help, always, no matter how much they have to do, they always come and help.* *Because I missed that when I worked hands-on at the health center, where you were more on your own and had very vague referrals that you had to try to solve.*	Have tried different ways of working during days Can plan their day to best suit their own needs and their own circumstances Is not solely responsible for the patient Having close contact with physicians Being physically close to them Helping each other across professional roles Appreciates the exchange with other professional groups	Utilizing degrees of freedom by collected knowledge Working autonomously Working in cross-professional teams

## 4 Findings

**Figure 1:**
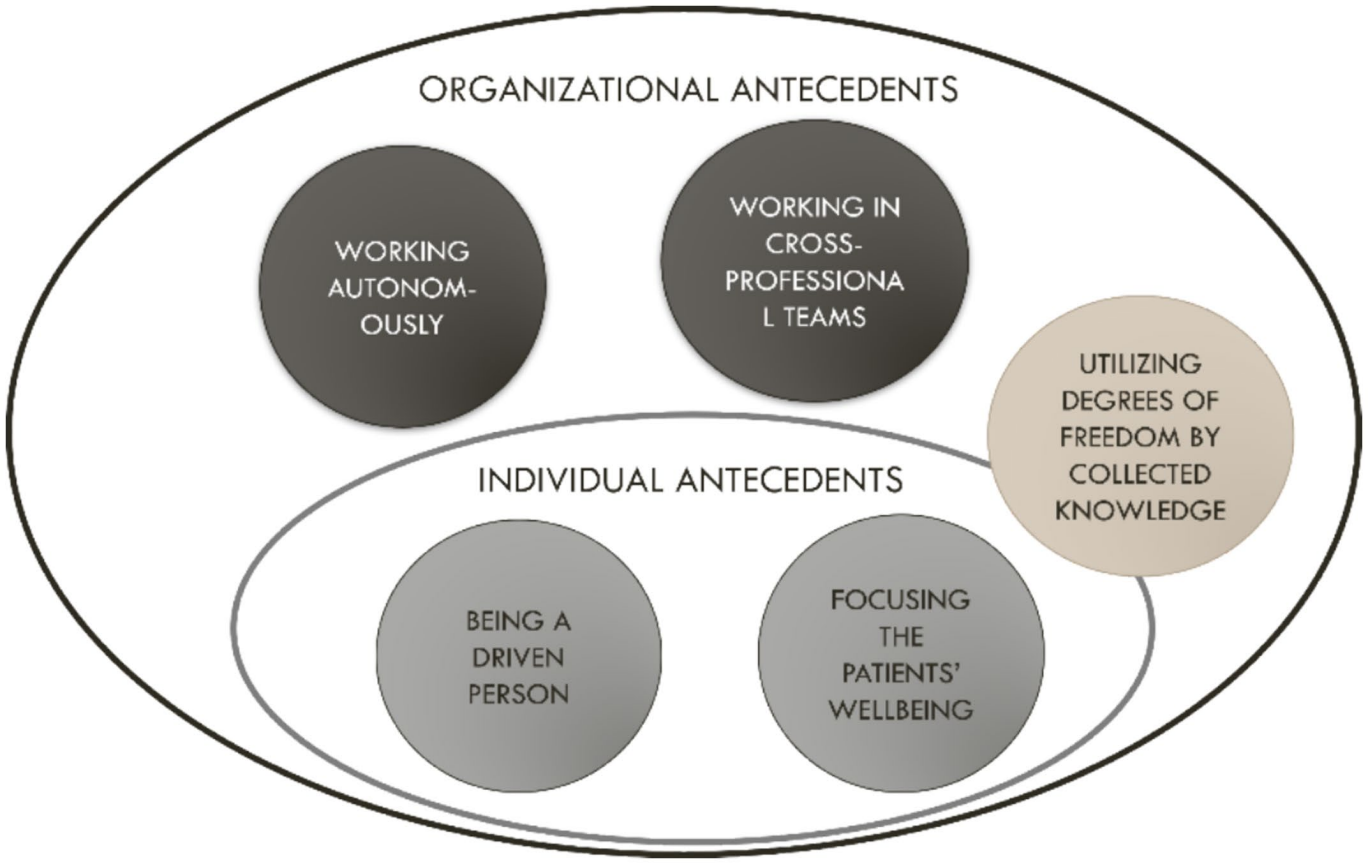
Summarizing and visualizing the findings: Health-promoting job crafting among healthcare employees was facilitated by individual drive, patient-centered values, experiential knowledge, and organizational conditions that enabled autonomy through supportive structures and access to professional freedom.

Antecedents of health-promoting job crafting among healthcare employees were identified on the individual level as well as in organizational structures. The most prominent antecedent for health-promoting job crafting among the employees in this study was that almost all of them considered themselves, or were interpreted, as driven people. Regardless of working in contexts with perceived support from colleagues and managers, or if work was mostly conducted on their own or in constraining work contexts, the employee respondents were adapting and changing work in a direction that promoted their well-being. Another individual antecedent was the employee respondents’ focus on the patient’s well-being: their intention to consider what was best for the patient was aligned with their crafting strategies, which increased the perceived meaningfulness in work. The third antecedent was being able to utilize degrees of freedom, which was gained through collected knowledge among the employees–for example, through experience, education, or from working in cross-disciplinary teams. Here, organizational-level conditions and structures were involved in enabling autonomy for health-promoting job crafting. The emerging antecedents are presented below and summarized in Figure 1.

### 4.1 Being a driven person

Employee respondents expressed a strong inner drive for personal development and managing what was–for them–the important work. They were proud of their professional identity, wanted to do a good job, and to develop personally and professionally. Most of them also said that they wanted to give something back and contribute to the organization.

The employees had different opinions about their work contexts. Some talked about a supportive climate and trustworthy management. Others said the organization of work was poor and that they had little support within the workgroup and from their closest manager. Regardless of context and support, most employees considered hindrances and demands in work as challenges, and they were not afraid to stand on their own as long as they reached their goals. One respondent said:

*I think it depends on the person. Because I am like that. I’m looking for a challenge. I want to do things … I want to … implement improvements. But if I do not get a response, if I do not get permission, then I’m not interested. Then I’ll move on, of course.*

One perception among the employees was that they worked ‘over and above their competence’, thus being challenged in their expertise, and crafted to learn new skills and to grow professionally, for example by asking for challenging tasks:

*So everyone is working at the forefront of their expertise. We can do everything we are educated in, and everything we have practical experience of.*

Some employees talked about being engaged and motivated, and some about the complexity of work as positively challenging. Younger and less experienced employees were keen to learn their jobs, and to become good in their new expertise. Job crafting strategies described by these respondents included mainly crafting their tasks and relations; they focused on learning and asked for more and developing responsibilities, and they also used their colleagues for advice and a kind of informal mentorship. With new competencies came more confidence and a greater understanding of the care conducted in their department.

### 4.2 Focusing on the patients’ wellbeing

Focusing on the patient’s well-being and doing the best possible for the patient was said to bring motivation and meaningfulness to work. Being there for the patients was considered a natural part of the job but was also said to add more meaning to the work. Employees explained how work was crafted by adapting and managing work with the patient’s best interest in mind:

*You have to remind yourself that it is their visit, it is not my visit. I do the professional part, but this is about them. They can control and decide as long as I do not break any boundaries.*

Employees emphasized focusing on the patient’s well-being in times of stress and staff shortages. They also took pride in knowing about all patients hospitalized in the department, for example, to be able to answer questions from relatives. Thus, respectful considerate treatment and flexibility were important, along with letting the patient’s circumstances set the agenda for the visit:

*I always think about whether I want my family here. I will take care of them as if it’s someone’s mother that I am taking care of, or someone’s daughter... I think it is about respect and that you want the same thing.*

Among some of the older and more experienced employees, work was no longer considered something they did to pursue a career and develop professionally. Even though they talked about a positive learning climate and development opportunities, they seemed more content with their work situation as it was and often mentioned caring for the patient, and keeping the focus on the patient as the most important and motivating during workdays, even when they did not enjoy work:

*You feel that you are helping someone, even if sometimes you have to do things that are not fun. At least we have done a good job.*

This focus on caring for the patients’ well-being was, however, also common among younger employees. Focusing on the patient seemed to precede job crafting strategies with the purpose of making the patient’s situation better, and making them feel safe and relaxed during treatment. This focus could ease the mind of the caregiver and add meaning to the work. Thus, focusing on the patients and their best interests seems to be one reason for healthcare professionals to craft their jobs.

### 4.3 Utilizing degrees of freedom by collected knowledge

The employees described working in complex organizations, with limited control and oversight to see opportunities and have the freedom for job crafting. This meant that their crafting was enabled by the autonomy that was built by experience or in their profession, or by the shared knowledge and created freedom that was built in well-functioning cross-professional teams. Thus, utilizing degrees of freedom in work through collected knowledge and comprehensiveness was an antecedent for their job crafting strategies. With autonomy and organized cross-professional teams came an ‘organizational mandate’ to craft at individual- and team levels. When utilizing perceived degrees of freedom, employee respondents saw opportunities to craft for professional development, as well as to manage challenges in better ways. For example, the employees sometimes asked for responsibilities outside their occupational roles and planned to meet their patients together with other professionals. Those strategies were said to contribute to development within their profession.

#### 4.3.1 Working autonomously

With formal autonomy in work, or the achieved autonomy through experience, employee respondents could, through their understanding, plan their working days and how to work with their patients. This freedom led to many crafting strategies; for example, they described how they developed templates in work–to adapt how to conduct assessments–as well as how they developed strategies to recover during workdays:

*I have tried to make two home visits a day but it does not really work, my head gets chaotic. If one is tough and demanding, you do not have the energy for another one in the afternoon and you never know what to expect, so it is not possible.*

Some respondents worked in more than one department, and the formal autonomy and experience in work enabled them to be available for their colleagues when needed, and to crafting strategies that saved time and energy as well as provided development opportunities. For example, they created structures for themselves and chose to plan their days together with colleagues in other departments to optimize their schedule as well as the workflow in the departments.

#### 4.3.2 Working in cross-professional teams

When autonomy was created through organized cross-professional teams, there was both formal and informal collaboration within and between care teams. Both managers and employees said that traditional hierarchical structures at work decreased; when working cross-professionally, the comments were that professional roles were less important. For example, one clinic manager said that *‘the dentist is no longer the star in the room’*, and dental- and assistant nurses were delegated more tasks than normally expected. From the managers’ perspective, one objective was to enable employees to grow within their current position, and to reduce the risk of losing important skills from experienced people moving on to other departments. One ward manager said:

*It is important to make sure that the workplace does not become a passage because a care department can easily be a thoroughfare if you want to proceed to surgery … But we have tried to create a workplace where you can work for 10 years and still feel that you have developed.*

To have a trustful and innovative climate within the work group was also considered important in terms of perceived freedom and opportunities among the employees:

*You feel that nothing is impossible [in the department]. That is exactly how I feel. Nothing is impossible!*

Working cross-professionally was said to be stimulating. When working close to others, and learning from each other when finding common solutions, employee respondents talked about a better understanding of their role in the patient’s care chain as well as having more tools to handle difficult situations. This way of working could still occur without being formally organized within the department since employees actively chose to work closely together with colleagues from other professional groups:

*I have close contact with the physicians here; they sit in the corridor next door. They help me a lot and I help them and so on. That exchange is great fun. And … you always get help, always, no matter how much they have to do, they always come and help.*

## 5 Discussion

The findings in this study identified antecedents of health-promoting job crafting among healthcare employees that can be related to SOC. The focused antecedents were on an individual level but also enabled making use of organizational structures, the perceived autonomy in professions and of teamwork. In the following, the results are analyzed through the lens of SOC and discussed in relation to earlier studies.

### 5.1 Job crafting and work-related sense of coherence in healthcare

**Figure 2:**
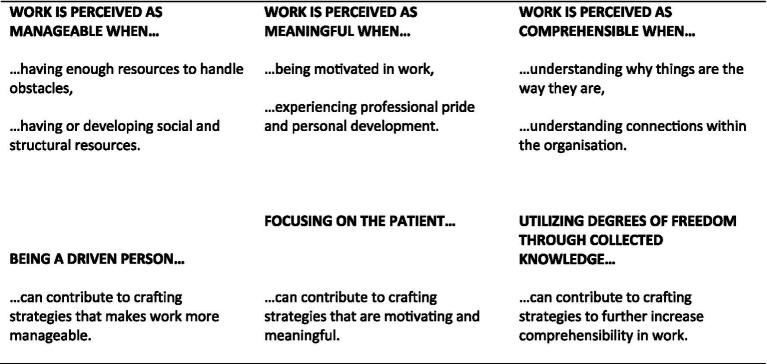
Proposed model of how the findings of antecedents for job crafting are related to a sense of coherence: Being a driven person as the basis for job crafting manageability in complex work; Focusing on the patient as the major antecedent to craft meaningfulness at work; and, Utilizing degrees of freedom as crucial for crafting comprehensibility at work.

The crafting strategies that the respondents chose to engage in were interpreted as increasing the employees’ work-related SOC, which is in line with the quantitative findings by Lichtenthaler and Fischbach (2016). The respondents were not asked specifically about the three components comprehensibility, manageability, and meaningfulness, but the way they described their job crafting strategies and the conditions that facilitated these strategies was connected to promoting their well-being through work being more comprehensible, manageable, and meaningful. Figure 2 summarizes how the findings in this study relate to a work-related SOC.

#### 5.1.1 Crafting for manageability in work

Being a driven person led to crafting strategies such as adjusting methods for assessments and planning patient activities closely together to facilitate work. Work is manageable when being able to understand and handle the context, and crafting strategies derived from this antecedent led to making work more manageable.

Overall, the respondents in this study were highly engaged in their work and had an inner drive for personal and professional development. These proactive characteristics have been identified as antecedents of job crafting (Bakker et al., 2012). In addition, employees in intensive work have previously been found to be highly engaged, despite the high workload and stress levels (Hakanen et al., 2019), and to master the intensity with salutogenic crafting strategies (Palm and Eriksson, 2018). Since work engagement is not only a consequence of job crafting but also an antecedent of individual job crafting within healthcare (Hakanen et al., 2018), highly engaged healthcare employees may find strategies to craft in a health-promoting manner despite stressful periods at work and limited possibilities.

In connection to being a driven person, some respondents emphasized support through mutual trust and strong social support at work. An innovative learning climate, where colleagues were open to new ideas, as well as managers who promoted this kind of environment were recurrent in the participating care departments, and were said to facilitate health-promoting job crafting. On the workplace level, people-oriented leadership approaches and organizational support have been found to promote job crafting and other actions taken to handle intensive work with health-promoting outcomes (Audenaert et al., 2020; Esteves and Pereira Lopes, 2017; Lazazzara et al., 2020; Palm and Eriksson, 2018; Pan et al., 2021). So too has a supportive climate among colleagues (Audenaert et al., 2020; Jutengren et al., 2020). There were, however, also respondents who described less supportive and non-trusting work contexts. Previous research indicates that a constraining work context derives avoidance oriented crafting, such as social reduction and withdrawal crafting, and, in extension, negative experiences of job crafting–for example, stress and overload (Lazazzara et al., 2020). Chang et al. (2020) found that nurses working in surgery were less prone to craft their jobs than nurses working in general departments, indicating that work settings have different effects on individuals’ job crafting and that some contexts may hinder job crafting. Access to important resources to shape the job is a recurrent job crafting antecedent (e.g., Tims and Bakker, 2010), and the level of autonomy as well as perceived intensity in work for nurses in surgery probably differ from those in general practice. The respondents in this study, however, crafted in approach-oriented ways (e.g., adding tasks and reframing roles) with health-promoting outcomes (e.g., meaningfulness and job satisfaction), regardless of their context: respondents working in surgery as well as in constraining social contexts described different motives and strategies for health-promoting job crafting. Being a driven person thus seemed to outweigh constraining working conditions; driven people seem to strive to craft in health-promoting manners regardless of working context. Recent findings found that job crafting strategies to re-organize work, and increased collaboration among healthcare professionals, was adapted by general practitioners during the Covid-19 pandemic, indicating that these strategies can increase manageability in constraining work conditions (Månsson Sandberg et al., 2024).

#### 5.1.2 Crafting for meaningfulness in work

Crafting strategies focusing on patients’ well-being was considered motivating and adding meaning to work among the respondents, thus increasing the perceived meaningfulness of work. Although the care of patients can be considered an obvious purpose in the healthcare sector, the way the respondents talked about caring for, and focusing on, the patients suggests that this also was a basic premise for *why* and *how* they crafted their jobs. Adjustments were constantly made to improve conditions for the patients; the respondents put the patients in control of the caring arrangements and crafted their jobs to fit as closely as possible between the patient’s needs and their own professional goals. In line with our findings, Månsson Sandberg et al. (2024) recently found similar meaning-making crafting strategies among general practionars during the COVID-19 pandemic. Their findings indicated that crafting for the common good and care of patients was the primary focus to increase meaning in work during this challenging period. Meaningfulness in terms of focusing on the patient can, according to earlier studies, be related to the concept of a calling. Bengtsson and Flisbäck (2017) outline five main components of a calling: (1) work appears as an external summons; (2) the purpose of the work activity is to serve a higher cause; (3) work is carried out using personality as a tool; (4) work involves self-sacrifice; and (5) work gives rise to elevation. Even though Bengtsson and Flisbäck (2017) emphasize that there is a ‘potential dark side of calling,’ such as being overworked, exploited, and making sacrifices, their studies demonstrate that the calling can, in fact, be a resource for well-being because the individual positions their work within a broader existential context. In the job crafting literature, an unanswered occupational calling has been identified as a job crafting antecedent, and engaging in job crafting can alter perceived meaning and purpose in work (Berg et al., 2008; Berg et al., 2010a; Wrzesniewski and Dutton, 2001). The respondents in this study did not express an unanswered calling; however, a professional calling within healthcare may be closely related to engaging in crafting strategies aligned with the calling. Thus the focus on the patients may be part of a professional calling, which in itself can motivate people to engage in crafting strategies to increase meaningfulness in work. Crafting for comprehensibility in work.

Utilizing degrees of freedom at work through collected knowledge contributed to a more comprehensible work situation when respondents were able to see their part in a larger whole. A more comprehensive picture of the care chain can increase comprehensibility in work (Antonovsky, 1987, 1996; Vaandrager and Koelen, 2013). Working in cross-professional teams gave respondents a comprehensive and more understandable picture of the patient’s care. This is in line with the findings of Nilsson et al. (2012), who found that “a more comprehensive picture of a patient’s care” was attained when planning and conducting work in close collaboration with other professionals (Nilsson et al., 2012, p. 161).

In addition, formal autonomy through education was shaping degrees of freedom in work. There was, however, no notable difference in the amount of job crafting activities between different professional groups. The qualitative differences lay more in *why* and *how* jobs were crafted: respondents with autonomy were able to craft the way their specific tasks (e.g., cognitive and physical assessments) were conducted and saw many opportunities to do so. Job autonomy has previously been found to be an important job crafting antecedent on the organizational level (Ghazzawi et al., 2021; Tims and Bakker, 2010), and higher-ranked employees with more formal autonomy tend to craft their work differently than lower-ranked employees (Berg et al., 2010b). In this study, respondents worked in organizational structures where traditional hierarchies were said to be blurred, and the perceived degrees of freedom that followed when working in cross-professional teams were said to facilitate job crafting strategies. Thus, perceived freedom and autonomy in work rather than traditional hierarchical ranks might be a better indicator of individual crafting within public healthcare. Crafting new roles (e.g., the specialized roles initiated and developed by some respondents) can be challenging in the healthcare sector, where daily care for the patients still is the main task (Felder et al., 2024). Autonomy in work can, however, facilitate crafting strategies to develop a both manageable and comprehensible role for the specialized professional roles.

Job crafting strategies may change due to work experience: more work experience can facilitate individual job crafting (Harbridge et al., 2022). In this study, work experience provided respondents with more perceived degrees of freedom to utilize in crafting strategies similarly to those with formal autonomy in work. Thus, experienced employees may find more ways to craft more manageable work in a restrictive work context, as previously indicated by Mayson and Bardoel (2021).

### 5.2 Methodological considerations and limitations

The respondents in this study were recruited from professional networks in the research group. The participating departments were chosen because of their previous and current efforts in developing their work environment. It must be considered that this sampling strategy may have provided informants working in departments that are non-typical for the public healthcare sector, where many managers struggle with a demanding work environment due to, for example, complex organizations and financial savings. However, we consider that this has provided important information about health-promoting job crafting and its antecedents that otherwise would not appear. As mentioned in the Method section, there were no employees working only or mostly night shifts among the informants, and those who occasionally worked night shift had their main affiliation among the day staff. Thus, the perspective of night shift workers’ job crafting, for whom facilitating and constraining conditions might differ from those working daytime, was not included in this study and is considered a limitation of the study design. This this is a research subject to study further.

Managers were involved in the data collection in terms of asking potential informants about their willingness to participate. This may have led to employees being selected from the managers’ convenience rather than from an open invitation to all employees in the department. In addition, we cannot fully rule out that employees participated in fear of being negatively affected by declining participation (Resnik, 2016). To ensure confidentiality to the greatest extent possible, interviews were conducted in private rooms, and no data was handled outside the research group. In the case of further contact being required, the managers provided the authors’ contact information to the respondents so that they could be contacted directly, for example in order to clarify questions.

The data collection took place over 2 years, which could have affected the dependability of the study design. It is however considered that data were collected during relatively similar conditions and before the outbreak of the COVID-19 pandemic. As mentioned above, the first author of this paper conducted the preliminary analysis. Thereafter both authors discussed and interpreted the data in an iterative and reflective process. The research and analysis design is considered to increase the quality and trustworthiness of the study (Graneheim et al., 2017; Stenfors et al., 2020).

### 5.3 Practical implications

Job crafting is, in general, positive for employees as well as for workgroups and workplaces, in terms of, for example, employee well-being and productivity (c.f. Felder et al., 2024; Lichtenthaler and Fischbach, 2016; Tims and Bakker, 2010; Wrzesniewski and Dutton, 2001). Since job crafting also seems to be related to a work-related SOC–which is a strong predictor of perceived health–it is suggested to promote health-promoting job crafting within public healthcare on both the individual and organizational levels. Some practical examples from the findings of this study of how to achieve this include to provide more autonomy whenever possible and enabling professional groups to collaborate. The latter can, however, be challenging in a working context still influenced to some extent by traditional hierarchies (Felder et al., 2024). A management approach that focus on trust, authenticity, and empowerment can be important to provide employees with greater autonomy and mandate to develop within their professional roles (Dellve and Eriksson, 2017; Wang et al., 2024). To experience more autonomy in work may contribute to employees crafting a more comprehensible and manageable work situation for themselves. According to the findings in this study, autonomy was perceived from both formal structures in the professional roles, and from experience and cross-professional workgroups.

Being able to craft their professional roles can motivate healthcare employees to stay with their current employer (Felder et al., 2024). However, it is suggested to let junior employees focus on their current tasks, and not assign them with more complex tasks, that might stop their job crafting–while more experienced employees can be assigned more complex, or additional, tasks to stimulate their job crafting (Wang et al., 2024). Meaningfulness in work seems to originate in the healthcare employees’ focus on their patient, and a potential perceived calling in work (Bengtsson and Flisbäck, 2017). Since personal factors–such as being a driven person, and focusing on the patient’s well-being in all parts of work–seem to facilitate health-promoting job crafting among healthcare employees, it is suggested to talk about those aspects in the workplace; for instance, during joint meetings or personal development dialogues between managers and employees.

## 6 Conclusion

The findings in this study suggest that facilitating antecedents of health-promoting job crafting can be found within workplace conditions, the workgroup culture, and the employees themselves, and that the antecedents interplay and interact within and between the different levels. Job crafting strategies derived from the identified antecedents, namely *being a driven person, focusing on the patient’s well-being,* and *utilizing degrees of freedom through collected knowledge*, were interpreted as being health-promoting in terms of contributing to a sense of manageability, meaningfulness, and comprehensibility in work. Firstly, to have an inner drive seems to outweigh poor organizational conditions when healthcare employees craft for their professional development and manageability in work. Secondly, having a focus on the patients’ best interest seems to precede meaning-making job crafting strategies. Finally, utilizing the perceived degrees of freedom in work can contribute to a more manageable and comprehensible work situation.

## Data Availability

The raw data supporting the conclusions of this article will be made available by the authors, without undue reservation.
